# Supplementary material for: Racism, homophobia, and the sexual health of young Black men who have sex with men in the United States: A systematic review
Source: PLoS One. 2025 Jan 16;20(1):e0316532. doi: 10.1371/journal.pone.0316532 (PMC11737682; doi:10.1371/journal.pone.0316532)
Supplement: S1 Appendix — (PDF) [file pone.0316532.s002.pdf]

S1 Appendix. Search strategies

Librarian searcher: Leila Ledbetter
Peer Reviewer: Samantha Kaplan, PhD, MLIS

Original Search Date: May 20, 2022
Update Search Date: March 13, 2023, August 15, 2023

Database: MEDLINE (PubMed)

Set #		Results May 20, 2022	Results March 13, 2022	Results August 15, 2023
1<br>Men	"Homosexuality, Male"[Mesh] OR "men having sex with men"[tiab] OR "men who have sex with men"[tiab] OR "men who have sex with other men"[tiab] OR MSM[tiab] OR BMSM[tiab] OR AAMSM[tiab] OR (("Male"[Mesh] OR "men"[Mesh:NoExp] OR male[tiab] OR males[tiab] OR men[tiab] OR man[tiab]) AND ("Bisexuality"[Mesh] OR bisexual*[tiab] OR homosexual*[tiab] OR gay[tiab] OR gays[tiab] OR LGBT[tiab] OR LGBTQ[tiab] OR LGBTI[tiab] OR LGBTQIA[tiab] OR GLBT[tiab] OR GLBTQ[tiab] OR nonheterosexual[tiab] OR "non-heterosexual"[tiab] OR "non heterosexuals"[tiab] OR nonheterosexuals[tiab] OR pansexual*[tiab] OR polysexual*[tiab] OR queer[tiab] OR "same sex"[tiab] OR "same-sex"[tiab] OR "sexual minorities"[tiab] OR "sexual minority"[tiab] OR "sexual orientation"[tiab]))	48,539	51,234	
2<br>Young adult	"Young Adult"[Mesh] OR "Adolescent"[Mesh] OR "Adolescent Health Services"[Mesh] OR "young adult"[tiab] OR "young adults"[tiab] OR "young adulthood"[tiab] OR AYA[tiab] OR adolescence[tiab] OR adolescent[tiab] OR adolescents[tiab] OR youth[tiab] OR youths[tiab] OR "emerging adult"[tiab] OR "emerging adults"[tiab] OR teen[tiab] OR teens[tiab] OR teenager[tiab] OR teenagers[tiab]	2,777,857	2,822,485	

3<br><br>Black	"African Americans"[Mesh] OR "African American"[tiab] OR "African Americans"[tiab] OR "African ancestry"[tiab] OR Black[tiab] OR Blacks[tiab] OR "minority group"[tiab] OR "minority groups"[mh] OR "minority groups"[tiab] OR "minority population"[tiab] OR "minority populations"[tiab] OR "minority health"[mh] OR "minority health"[tiab] OR minority[tiab] OR minorities[tiab] OR marginalize[tiab] OR marginalized[tiab] OR marginalizing[tiab] OR marginalise[tiab] OR marginalised[tiab] OR marginalizing[tiab] OR oppressed[tiab] OR BIPOC[tiab] OR underserved[tiab] OR disadvantaged[tiab] OR "afro-american"[tiab] OR "Afro-Caribbean"[tiab] OR "Black Caribbean"[tiab] OR "African Caribbean"[tiab] OR "Black West Indian"[tiab] OR "Afro West Indian"[tiab] OR "Black Antillean"[tiab] OR "Afro Antillean"[tiab] OR "African diaspora"[tiab] OR "Black Creole"[tiab] OR ((Jamaica[tiab] OR Jamaican[tiab] OR Haiti[tiab] OR Haitian[tiab] OR Bahamas[tiab] OR Bahamian[tiab] OR Barbados[tiab] OR Barbadian[tiab] OR Bermuda[tiab] OR Bermudian[tiab] OR Dominican[tiab] OR Grenada[tiab] OR Grenadian[tiab] OR Kitts[tiab] OR Kittian[tiab] OR Nevis[tiab] OR Nevisian[tiab] OR Lucia[tiab] OR Lucian[tiab] OR Surinam[tiab] OR Surinamese[tiab] OR Trinidad[tiab] OR Trinidadians[tiab] OR Tobago[tiab] OR Tobagonians[tiab] OR Vincent[tiab] OR Vincentian[tiab]) AND (America[tiab] OR American[tiab] OR Americans[tiab] OR Afro[tiab] OR african[tiab]))	338,791	347,947	
4<br><br>Discrimination<br><br>Racism	"Systemic Racism"[Mesh] OR "Homophobia"[Mesh] OR "Masculinity"[Mesh] OR prejudice[mesh] OR "social discrimination"[mesh] OR stereotyping[mesh] OR "social	2,254,815	2,359,577	

Homophobia	perception"[mesh] OR Racism[tiab] OR racisms[tiab] OR Racial[tiab] OR prejudice[tiab] OR prejudiced[tiab] OR prejudicing[tiab] OR prejudices[tiab] OR discriminate[tiab] OR discriminates[tiab] OR discriminated[tiab] OR discrimination[tiab] OR discriminating[tiab] OR "social perceptions"[tiab] OR "social perception"[tiab] OR "social identity"[tiab] OR "social identities"[tiab] OR isolation[tiab] OR isolate[tiab] OR isolates[tiab] OR isolating[tiab] OR isolated[tiab] OR mistreat[tiab] OR mistreats[tiab] OR mistreated[tiab] OR alienate[tiab] OR alienates[tiab] OR alienated[tiab] OR mistreatment[tiab] OR alienation[tiab] OR homophobia[tiab] OR homophobic[tiab] OR masculine[tiab] OR masculinities[tiab] OR masculinity[tiab] OR perception[tiab] OR perceptions[tiab] OR bias[tiab] OR microaggression[tiab] or microaggressions[tiab] OR “homonegativity”[tiab]			
5<br><br>Sexual health	"Sexual Health"[Mesh] OR "Sexual Behavior"[Mesh] OR "Sex Education"[Mesh] OR "Sexually Transmitted Diseases"[Mesh] OR "contraception behavior"[MeSH] OR "Condoms"[Mesh] OR "Contraceptive Agents, Male"[Mesh] OR "Contraceptive Devices, Male"[Mesh] OR sex[tiab] OR sexual[tiab] OR sexuality[tiab] OR sexualities[tiab] OR sexually[tiab] OR intercourse[tiab] OR "Sexually Transmitted Diseases"[tiab] OR "Sexually Transmitted Disease"[tiab] OR "Sexually Transmitted Infections"[tiab] OR "Sexually Transmitted Infection"[tiab] OR STD[tiab] OR STDs[tiab] OR STI[tiab] OR STIs[tiab] OR "Venereal Diseases"[tiab] OR "Venereal Disease"[tiab] OR contraception[tiab] OR contraceptive[tiab] OR contraceptives[tiab] OR condom[tiab] OR condoms[tiab] OR "sexual health"[tiab] OR chancroid[tiab] OR chlamydia[tiab] OR chancroids[tiab] OR gonorrhea[tiab] OR "granuloma inguinale"[tiab] OR syphilis[tiab] OR "genital herpes"[tiab] OR "genitalis	2,315,505	2,450,363	

	herpes"[tiab] OR "Condylomata Acuminata"[tiab] OR "genital warts"[tiab] OR "genital wart"[tiab] OR "venereal warts"[tiab] OR "venereal wart"[tiab] OR HIV[tiab] OR "human immunodeficiency virus"[tiab] OR AIDS[tiab] OR "acquired immunodeficiency syndrome"[tiab] OR "pre-exposure prophylaxis" OR "PrEP" OR "health care"[tiab] OR "screening"[tiab]			
6	#1 AND #2 AND #3 AND #4 AND #5	1,333	1,398	
7	#6 NOT (Editorial[ptyp] OR Letter[ptyp] OR Comment[ptyp])	1,333	1,398	
8	#7 NOT (animals[MeSH Terms] NOT humans[MeSH Terms])	1,333	1,398	
9	#8 AND ("2022/05/01"[Date - MeSH] : "3000"[Date - MeSH])		88	
10	#8 AND ("2023/01/01"[Date - MeSH] : "3000"[Date - MeSH])			58

Database: CINAHL Complete (EBSCOhost)

Set #		Results May 20, 2022	Results March 13, 2022	Results August 15, 2023
1<br>Men	MH "Men Who Have Sex With Men" OR TI("men having sex with men" OR "men who have sex with men" OR "men who have sex with other men" OR MSM OR BMSM OR AAMSM) OR AB("men having sex with men" OR "men who have sex with men" OR "men who have sex with other men" OR MSM OR BMSM OR AAMSM)	8,671	9,214	9,384
2	MH "Male" OR MH "Men" OR TI(male OR males OR men OR man) OR AB(male OR males OR men OR man)	1,969,073	2,029,807	2,045,543
3	MH "Gay Men" OR MH "Bisexuality" OR TI(bisexual* OR homosexual* OR gay OR gays OR LGBT OR LGBTQ OR LGBTI OR LGBTQIA OR GLBT OR GLBTQ OR nonheterosexual OR "non-heterosexual" OR "non heterosexuals" OR nonheterosexuals OR pansexual* OR polysexual* OR queer OR	20,811	22,282	22,542

	"same sex" OR "same-sex" OR "sexual minorities" OR "sexual minority" OR "sexual orientation") OR AB(bisexual* OR homosexual* OR gay OR gays OR LGBT OR LGBTQ OR LGBTI OR LGBTQIA OR GLBT OR GLBTQ OR nonheterosexual OR "non-heterosexual" OR "non heterosexuals" OR nonheterosexuals OR pansexual* OR polysexual* OR queer OR "same sex" OR "same-sex" OR "sexual minorities" OR "sexual minority" OR "sexual orientation")			
4	S2 AND S3	13,448	14,265	14,458
5	S1 OR S4	19,066	20,283	20,611
6<br>Young adult	MH "Young Adult" OR MH "Adolescence+" OR MH "Adolescent Health" OR MH "Adolescent Health Services" OR TI("young adult" OR "young adults" OR "young adulthood" OR AYA OR adolescence OR adolescent OR adolescents OR youth OR youths OR "emerging adult" OR "emerging adults" OR teen OR teens OR teenager OR teenagers) OR AB("young adult" OR "young adults" OR "young adulthood" OR AYA OR adolescence OR adolescent OR adolescents OR youth OR youths OR "emerging adult" OR "emerging adults" OR teen OR teens OR teenager OR teenagers)	769,001	798,108	805,024
7<br>Black	MH "Black Persons" OR MH "Minority Groups" OR TI("minority groups" OR "minority population" OR "minority populations" OR "African American" OR "African Americans" OR "African ancestry" OR Black OR Blacks OR "minority group" OR "minority health" OR minority OR minorities OR marginalize OR marginalized OR marginalizing OR marginalise OR marginalised OR marginalizing OR oppressed OR BIPOC OR underserved OR disadvantaged OR "afro-american" OR "Afro-Caribbean" OR "Black Caribbean" OR "African Caribbean" OR "Black West Indian" OR "Afro West Indian" OR "Black Antillean"	148,596	157,755	160,002

	OR "Afro Antillean" OR "African diaspora" OR "black Creole") OR AB("minority groups" OR "minority population" OR "minority populations" OR "African American" OR "African Americans" OR "African ancestry" OR Black OR Blacks OR "minority group" OR "minority health" OR minority OR minorities OR marginalize OR marginalized OR marginalizing OR marginalise OR marginalised OR marginalizing OR oppressed OR BIPOC OR underserved OR disadvantaged OR "afro-american" OR "Afro-Caribbean" OR "Black Caribbean" OR "African Caribbean" OR "Black West Indian" OR "Afro West Indian" OR "Black Antillean" OR "Afro Antillean" OR "African diaspora" OR "black Creole")			
8	TI(Jamaica OR Jamaican OR Haiti OR Haitian OR Bahamas OR Bahamian OR Barbados OR Barbadian OR Bermuda OR Bermudian OR Dominican OR Grenada OR Grenadian OR Kitts OR Kittian OR Nevis OR Nevisian OR Lucia OR Lucian OR Surinam OR Surinamese OR Trinidad OR Trinidadians OR Tobago OR Tobagonians OR Vincent OR Vincentian) OR AB(Jamaica OR Jamaican OR Haiti OR Haitian OR Bahamas OR Bahamian OR Barbados OR Barbadian OR Bermuda OR Bermudian OR Dominican OR Grenada OR Grenadian OR Kitts OR Kittian OR Nevis OR Nevisian OR Lucia OR Lucian OR Surinam OR Surinamese OR Trinidad OR Trinidadians OR Tobago OR Tobagonians OR Vincent OR Vincentian)	7,026	7,302	7,239
9	TI(America OR American OR Americans OR Afro OR African) OR AB(America OR American OR Americans OR Afro OR African)	239,010	249,614	248,686
10	S8 AND S9	1,078	1,140	1,142

11	S7 OR S10	149,312	158,515	160,757
12<br>Discrimination<br>Racism<br>Homophobia	MH "Racism" OR MH "Homophobia" OR MH "Prejudice" OR MH "Masculinity" OR MH "Stereotyping" OR TI(Racism OR racisms OR Racial OR prejudice OR prejudiced OR prejudicing OR prejudices OR discriminate OR discriminates OR discriminated OR discrimination OR discriminating OR "social perceptions" OR "social perception" OR "social identity" OR "social identities" OR isolation OR isolate OR isolates OR isolating OR isolated OR mistreat OR mistreats OR mistreated OR alienate OR alienates OR alienated OR mistreatment OR alienation OR homophobia OR homophobic OR masculine OR masculinities OR masculinity OR perception OR perceptions OR bias OR microaggression or microaggressions OR homonegativity) OR AB(Racism OR racisms OR Racial OR prejudice OR prejudiced OR prejudicing OR prejudices OR discriminate OR discriminates OR discriminated OR discrimination OR discriminating OR "social perceptions" OR "social perception" OR "social identity" OR "social identities" OR isolation OR isolate OR isolates OR isolating OR isolated OR mistreat OR mistreats OR mistreated OR alienate OR alienates OR alienated OR mistreatment OR alienation OR homophobia OR homophobic OR masculine OR masculinities OR masculinity OR perception OR perceptions OR bias OR microaggression or microaggressions OR homonegativity)	390,267	417,336	425,827
13<br>Sexual health	MH "Sexual Health" OR MH "Contraceptive Agents, Male" OR MH "Contraceptive Devices+" OR MH "Sexual Behavior+" OR MH "Sex Education" OR MH "Sexually Transmitted Diseases+" OR MH "Condoms+" OR TI(sex OR sexual OR sexuality OR	745,846	785,331	789,919

	sexualities OR sexually OR intercourse OR "Sexually Transmitted Diseases" OR "Sexually Transmitted Disease" OR "Sexually Transmitted Infections" OR "Sexually Transmitted Infection" OR STD OR STDs OR STI OR STIs OR "Venereal Diseases" OR "Venereal Disease" OR contraception OR contraceptive OR contraceptives OR condom OR condoms OR "sexual health" OR chancroid OR chlamydia OR chancroids OR gonorrhea OR "granuloma inguinale" OR syphilis OR "genital herpes" OR "genitalis herpes" OR "Condylomata Acuminata" OR "genital warts" OR "genital wart" OR "venereal warts" OR "venereal wart" OR HIV OR "human immunodeficiency virus" OR AIDS OR "acquired immunodeficiency syndrome" OR "pre-exposure prophylaxis" OR "PrEP" OR "health care" OR "screening") OR AB(sex OR sexual OR sexuality OR sexualities OR sexually OR intercourse OR "Sexually Transmitted Diseases" OR "Sexually Transmitted Disease" OR "Sexually Transmitted Infections" OR "Sexually Transmitted Infection" OR STD OR STDs OR STI OR STIs OR "Venereal Diseases" OR "Venereal Disease" OR contraception OR contraceptive OR contraceptives OR condom OR condoms OR "sexual health" OR chancroid OR chlamydia OR chancroids OR gonorrhea OR "granuloma inguinale" OR syphilis OR "genital herpes" OR "genitalis herpes" OR "Condylomata Acuminata" OR "genital warts" OR "genital wart" OR "venereal warts" OR "venereal wart" OR HIV OR "human immunodeficiency virus" OR AIDS OR "acquired immunodeficiency syndrome" OR "pre-exposure prophylaxis" OR "PrEP" OR "health care" OR "screening")			
14	S5 AND S6 AND S11 AND S12 AND S13	608	665	689
15	Limiters - Research Article; Peer Reviewed	495	544	564
16	Limiters - Published Date: 20220501-20230331		49	
17	Limiters - Date: 20230301-			20

Database: APA PsycINFO (EBSCOhost)

Set #		Results May 20, 2022	Results March 13, 2022	Results August 15, 2023
1<br><br>Men	TI("men having sex with men" OR "men who have sex with men" OR "men who have sex with other men" OR MSM OR BMSM OR AAMSM) OR AB("men having sex with men" OR "men who have sex with men" OR "men who have sex with other men" OR MSM OR BMSM OR AAMSM)	5,942	6,251	6,378
2	DE "Human Males" OR TI(male OR males OR men OR man) OR AB(male OR males OR men OR man)	585,984	600,251	607,965
3	DE "Same Sex Intercourse" OR DE "Male Homosexuality" OR DE "Bisexuality" OR TI(bisexual* OR homosexual* OR gay OR gays OR LGBT OR LGBTQ OR LGBTI OR LGBTQIA OR GLBT OR GLBTQ OR nonheterosexual OR "non-heterosexual" OR "non heterosexuals" OR nonheterosexuals OR pansexual* OR polysexual* OR queer OR "same sex" OR "same-sex" OR "sexual minorities" OR "sexual minority" OR "sexual orientation") OR AB(bisexual* OR homosexual* OR gay OR gays OR LGBT OR LGBTQ OR LGBTI OR LGBTQIA OR GLBT OR GLBTQ OR nonheterosexual OR "non-heterosexual" OR "non heterosexuals" OR nonheterosexuals OR pansexual* OR polysexual* OR queer OR "same sex" OR "same-sex" OR "sexual minorities" OR "sexual minority" OR "sexual orientation")	57,930	60,136	61,463
4	S2 AND S3	27,865	28,838	29,366
5	S1 OR S4	29,068	30,092	30,616
6<br><br>Young adult	DE "Emerging Adulthood" OR DE "Adolescent Health" OR TI("young adult" OR "young adults" OR "young adulthood" OR AYA OR adolescence OR adolescent OR adolescents OR youth OR youths OR "emerging adult" OR "emerging adults" OR teen OR teens OR teenager OR teenagers) OR AB("young adult" OR "young adults" OR "young adulthood" OR AYA OR adolescence OR adolescent OR	370,179	384,717	393,122

	adolescents OR youth OR youths OR "emerging adult" OR "emerging adults" OR teen OR teens OR teenager OR teenagers)			
7<br>Black	DE "Blacks" OR DE "Minority Groups" OR TI("minority groups" OR "minority population" OR "minority populations" OR "African American" OR "African Americans" OR "African ancestry" OR Black OR Blacks OR "minority group" OR "minority health" OR minority OR minorities OR marginalize OR marginalized OR marginalizing OR marginalise OR marginalised OR marginalizing OR oppressed OR BIPOC OR underserved OR disadvantaged OR "afro-american" OR "Afro-Caribbean" OR "Black Caribbean" OR "African Caribbean" OR "Black West Indian" OR "Afro West Indian" OR "Black Antillean" OR "Afro Antillean" OR "African diaspora" OR "black Creole") OR AB("minority groups" OR "minority population" OR "minority populations" OR "African American" OR "African Americans" OR "African ancestry" OR Black OR Blacks OR "minority group" OR "minority health" OR minority OR minorities OR marginalize OR marginalized OR marginalizing OR marginalise OR marginalised OR marginalizing OR oppressed OR BIPOC OR underserved OR disadvantaged OR "afro-american" OR "Afro-Caribbean" OR "Black Caribbean" OR "African Caribbean" OR "Black West Indian" OR "Afro West Indian" OR "Black Antillean" OR "Afro Antillean" OR "African diaspora" OR "black Creole")	192,455	201,169	206,202
8	TI(Jamaica OR Jamaican OR Haiti OR Haitian OR Bahamas OR Bahamian OR Barbados OR Barbadian OR Bermuda OR Bermudian OR Dominican OR Grenada OR Grenadian OR Kitts OR Kittian OR Nevis OR Nevisian OR Lucia OR Lucian OR Surinam OR Surinamese OR Trinidad OR Trinidadians OR Tobago OR Tobagonians OR Vincent OR Vincentian) OR AB(Jamaica OR Jamaican OR Haiti OR Haitian OR Bahamas OR Bahamian OR Barbados OR Barbadian OR Bermuda OR Bermudian OR Dominican OR Grenada OR Grenadian OR Kitts OR Kittian OR Nevis OR Nevisian OR	7,033	7,301	7,416

	Lucia OR Lucian OR Surinam OR Surinamese OR Trinidad OR Trinidadians OR Tobago OR Tobagonians OR Vincent OR Vincentian)			
9	TI(America OR American OR Americans OR Afro OR African) OR AB(America OR American OR Americans OR Afro OR African)	232,322	238,787	242,452
10	S8 AND S9	1,339	1,393	1,424
11	S7 OR S10	193,218	201,964	207,016
12<br>Discrimination<br>Racism<br>Homophobia	DE "Racism" OR DE "Systemic Racism" OR DE "Homosexuality (Attitudes Toward)" OR DE "Prejudice" OR DE "Masculinity" OR DE "Stereotyped Attitudes" OR TI(Racism OR racisms OR Racial OR prejudice OR prejudiced OR prejudicing OR prejudices OR discriminate OR discriminates OR discriminated OR discrimination OR discriminating OR "social perceptions" OR "social perception" OR "social identity" OR "social identities" OR isolation OR isolate OR isolates OR isolating OR isolated OR mistreat OR mistreats OR mistreated OR alienate OR alienates OR alienated OR mistreatment OR alienation OR homophobia OR homophobic OR masculine OR masculinities OR masculinity OR perception OR perceptions OR bias OR microaggression or microaggressions OR homonegativity) OR AB(Racism OR racisms OR Racial OR prejudice OR prejudiced OR prejudicing OR prejudices OR discriminate OR discriminates OR discriminated OR discrimination OR discriminating OR "social perceptions" OR "social perception" OR "social identity" OR "social identities" OR isolation OR isolate OR isolates OR isolating OR isolated OR mistreat OR mistreats OR mistreated OR alienate OR alienates OR alienated OR mistreatment OR alienation OR homophobia OR homophobic OR masculine OR masculinities OR masculinity OR perception OR perceptions OR bias OR	648,239	674,045	688,522

	microaggression or microaggressions OR homonegativity)			
13<br><br>Sexual health	DE "Sexual Health" OR DE "Contraceptive Devices" OR DE "Psychosexual Behavior" OR DE "Sex Education" OR DE "Sexually Transmitted Diseases" OR DE "Condoms" OR TI(sex OR sexual OR sexuality OR sexualities OR sexually OR intercourse OR "Sexually Transmitted Diseases" OR "Sexually Transmitted Disease" OR "Sexually Transmitted Infections" OR "Sexually Transmitted Infection" OR STD OR STDs OR STI OR STIs OR "Venereal Diseases" OR "Venereal Disease" OR contraception OR contraceptive OR contraceptives OR condom OR condoms OR "sexual health" OR chancroid OR chlamydia OR chancroids OR gonorrhea OR "granuloma inguinale" OR syphilis OR "genital herpes" OR "genitalis herpes" OR "Condylomata Acuminata" OR "genital warts" OR "genital wart" OR "venereal warts" OR "venereal wart" OR HIV OR "human immunodeficiency virus" OR AIDS OR "acquired immunodeficiency syndrome" OR "pre-exposure prophylaxis" OR "PrEP" OR "health care" OR "screening") OR AB(sex OR sexual OR sexuality OR sexualities OR sexually OR intercourse OR "Sexually Transmitted Diseases" OR "Sexually Transmitted Disease" OR "Sexually Transmitted Infections" OR "Sexually Transmitted Infection" OR STD OR STDs OR STI OR STIs OR "Venereal Diseases" OR "Venereal Disease" OR contraception OR contraceptive OR contraceptives OR condom OR condoms OR "sexual health" OR chancroid OR chlamydia OR chancroids OR gonorrhea OR "granuloma inguinale" OR syphilis OR "genital herpes" OR "genitalis herpes" OR "Condylomata Acuminata" OR "genital warts" OR "genital wart" OR "venereal warts" OR "venereal wart" OR HIV OR "human immunodeficiency virus" OR AIDS OR "acquired immunodeficiency syndrome" OR "pre-exposure prophylaxis" OR "PrEP" OR "health care" OR "screening")	548,094	567,117	577,612
14	S5 AND S6 AND S11 AND S12 AND S13	281	306	318
15	Limiters – Academic journals	213	234	
16	Limiters - Published Date: 20220501-20230331		20	

17	Limiters - Publication Type: Peer Reviewed Journal			241
18				15

Database: Sociology Source Ultimate (EBSCOhost)

Set #		Results May 20, 2022	Results March 13, 2022	Results August 15, 2023
1<br>Men	DE "MEN who have sex with men" OR TI("men having sex with men" OR "men who have sex with men" OR "men who have sex with other men" OR MSM OR BMSM OR AAMSM) OR AB("men having sex with men" OR "men who have sex with men" OR "men who have sex with other men" OR MSM OR BMSM OR AAMSM)	1,672	1,760	1,763
2	DE "Men" OR TI(male OR males OR men OR man) OR AB(male OR males OR men OR man)	170,454	178,737	181,152
3	DE "Homosexuality" OR DE "Bisexuality" OR TI(bisexual* OR homosexual* OR gay OR gays OR LGBT OR LGBTQ OR LGBTI OR LGBTQIA OR GLBT OR GLBTQ OR nonheterosexual OR "non-heterosexual" OR "non heterosexuals" OR nonheterosexuals OR pansexual* OR polysexual* OR queer OR "same sex" OR "same-sex" OR "sexual minorities" OR "sexual minority" OR "sexual orientation") OR AB(bisexual* OR homosexual* OR gay OR gays OR LGBT OR LGBTQ OR LGBTI OR LGBTQIA OR GLBT OR GLBTQ OR nonheterosexual OR "non-heterosexual" OR "non heterosexuals" OR nonheterosexuals OR pansexual* OR polysexual* OR queer OR "same sex" OR "same-sex" OR "sexual minorities" OR "sexual minority" OR "sexual orientation")	34,055	35,969	36,644
4	S2 AND S3	11,401	11,973	12,142
5	S1 OR S4	12,349	12,966	13,132
6	DE "Young Adults" OR DE "TEENAGERS" OR DE "ADOLESCENCE" OR DE "Adolescent	138,463	153,415	156,276

Young adult	Health" OR TI("young adult" OR "young adults" OR "young adulthood" OR AYA OR adolescence OR adolescent OR adolescents OR youth OR youths OR "emerging adult" OR "emerging adults" OR teen OR teens OR teenager OR teenagers) OR AB("young adult" OR "young adults" OR "young adulthood" OR AYA OR adolescence OR adolescent OR adolescents OR youth OR youths OR "emerging adult" OR "emerging adults" OR teen OR teens OR teenager OR teenagers)			
7<br>Black	DE "Black people" OR DE "PEOPLE of color" OR TI("minority groups" OR "minority population" OR "minority populations" OR "African American" OR "African Americans" OR "African ancestry" OR Black OR Blacks OR "minority group" OR "minority health" OR minority OR minorities OR marginalize OR marginalized OR marginalizing OR marginalise OR marginalised OR marginalizing OR oppressed OR BIPOC OR underserved OR disadvantaged OR "afro-american" OR "Afro-Caribbean" OR "Black Caribbean" OR "African Caribbean" OR "Black West Indian" OR "Afro West Indian" OR "Black Antillean" OR "Afro Antillean" OR "African diaspora" OR "black Creole") OR AB("minority groups" OR "minority population" OR "minority populations" OR "African American" OR "African Americans" OR "African ancestry" OR Black OR Blacks OR "minority group" OR "minority health" OR minority OR minorities OR marginalize OR marginalized OR marginalizing OR marginalise OR marginalised OR marginalizing OR oppressed OR BIPOC OR underserved OR disadvantaged OR "afro-american" OR "Afro-Caribbean" OR "Black Caribbean" OR "African Caribbean" OR "Black West Indian" OR "Afro West Indian" OR "Black Antillean" OR "Afro Antillean" OR "African diaspora" OR "black Creole")	132,052	139,965	142,353
8	TI(Jamaica OR Jamaican OR Haiti OR Haitian OR Bahamas OR Bahamian OR Barbados OR Barbadian OR Bermuda OR Bermudian OR Dominican OR Grenada OR	8,536	8,924	9,028

	Grenadian OR Kitts OR Kittian OR Nevis OR Nevisian OR Lucia OR Lucian OR Surinam OR Surinamese OR Trinidad OR Trinidadians OR Tobago OR Tobagonians OR Vincent OR Vincentian) OR AB(Jamaica OR Jamaican OR Haiti OR Haitian OR Bahamas OR Bahamian OR Barbados OR Barbadian OR Bermuda OR Bermudian OR Dominican OR Grenada OR Grenadian OR Kitts OR Kittian OR Nevis OR Nevisian OR Lucia OR Lucian OR Surinam OR Surinamese OR Trinidad OR Trinidadians OR Tobago OR Tobagonians OR Vincent OR Vincentian)			
9	TI(America OR American OR Americans OR Afro OR African) OR AB(America OR American OR Americans OR Afro OR African)	262,103	270,602	273,207
10	S8 AND S9	1,595	1,668	1,694
11	S7 OR S10	133,213	141,171	143,571
12<br>Discrimination<br>Racism<br>Homophobia	DE "Racism" OR DE "INSTITUTIONAL racism" OR DE "Homophobia" OR DE "Prejudices" OR DE "Masculinity" OR DE "Stereotypes" OR TI(Racism OR racisms OR Racial OR prejudice OR prejudiced OR prejudicing OR prejudices OR discriminate OR discriminates OR discriminated OR discrimination OR discriminating OR "social perceptions" OR "social perception" OR "social identity" OR "social identities" OR isolation OR isolate OR isolates OR isolating OR isolated OR mistreat OR mistreats OR mistreated OR alienate OR alienates OR alienated OR mistreatment OR alienation OR homophobia OR homophobic OR masculine OR masculinities OR masculinity OR perception OR perceptions OR bias OR microaggression or microaggressions OR homonegativity) OR AB(Racism OR racisms OR Racial OR prejudice OR prejudiced OR prejudicing OR prejudices OR discriminate OR discriminates OR discriminated OR	221,551	237,239	242,143

	discrimination OR discriminating OR "social perceptions" OR "social perception" OR "social identity" OR "social identities" OR isolation OR isolate OR isolates OR isolating OR isolated OR mistreat OR mistreats OR mistreated OR alienate OR alienates OR alienated OR mistreatment OR alienation OR homophobia OR homophobic OR masculine OR masculinities OR masculinity OR perception OR perceptions OR bias OR microaggression or microaggressions OR homonegativity)			
13<br><br>Sexual health	DE "Sexual Health" OR DE "MALE contraceptives" OR DE "HUMAN sexuality" OR DE "Sex Education" OR DE "Sexually Transmitted Diseases" OR DE "Condoms" OR TI(sex OR sexual OR sexuality OR sexualities OR sexually OR intercourse OR "Sexually Transmitted Diseases" OR "Sexually Transmitted Disease" OR "Sexually Transmitted Infections" OR "Sexually Transmitted Infection" OR STD OR STDs OR STI OR STIs OR "Venereal Diseases" OR "Venereal Disease" OR contraception OR contraceptive OR contraceptives OR condom OR condoms OR "sexual health" OR chancroid OR chlamydia OR chancroids OR gonorrhea OR "granuloma inguinale" OR syphilis OR "genital herpes" OR "genitalis herpes" OR "Condylomata Acuminata" OR "genital warts" OR "genital wart" OR "venereal warts" OR "venereal wart" OR HIV OR "human immunodeficiency virus" OR AIDS OR "acquired immunodeficiency syndrome" OR "pre-exposure prophylaxis" OR "PrEP" OR "health care" OR "screening") OR AB(sex OR sexual OR sexuality OR sexualities OR sexually OR intercourse OR "Sexually Transmitted Diseases" OR "Sexually Transmitted Disease" OR "Sexually Transmitted Infections" OR "Sexually Transmitted Infection" OR STD OR STDs OR STI OR STIs OR "Venereal Diseases" OR "Venereal Disease" OR contraception OR contraceptive OR contraceptives OR condom OR condoms OR "sexual health" OR chancroid OR chlamydia OR chancroids OR gonorrhea OR "granuloma inguinale" OR syphilis OR "genital herpes" OR "genitalis herpes" OR "Condylomata Acuminata" OR "genital warts" OR "genital wart" OR "venereal warts" OR "venereal wart" OR HIV	211,224	222,456	224,363

	OR "human immunodeficiency virus" OR AIDS OR "acquired immunodeficiency syndrome" OR "pre-exposure prophylaxis" OR "PrEP" OR "health care" OR "screening")			
14	S5 AND S6 AND S11 AND S12 AND S13	91	100	104
15	Limiters – Academic journals	77	86	
16	Limiters - Published Date: 20220501-20230331		6	
17	Limiters - Scholarly (Peer Reviewed) Journals			91
18	Limiters - Published Date: 20230301-			4
